# Nutrition fact label (NFL) use is related to meeting the requirements for vitamins and minerals not listed on NFLs: Data from the Korea National Health and Nutritional Examination Survey VIII (2019–2021) for the prepandemic and pandemic periods

**DOI:** 10.1371/journal.pone.0296268

**Published:** 2023-12-28

**Authors:** Jung Min Cho

**Affiliations:** K-Food Industry Research Institute, College of Culture and Tourism, Jeonju University, Jeonju-si, Republic of Korea; Federal University of ABC, BRAZIL

## Abstract

The credibility and wide usability of nutrition fact labels (NFLs) have increased due to the pandemic, which may lead to healthier nutritional choices. This cross-sectional study aimed to evaluate the association between NFL use and meeting the estimated average requirements (EARs) for vitamins and minerals not listed on NFLs during the prepandemic (2019, *n* = 6606) and pandemic periods (2020 and 2021, *n* = 12085) using KNHANES data. Household surveys, health behavior interviews, and health checkup examinations were conducted for all participants. Subjects were included in the unAware+noUse, Aware+noUse, and Aware+Use groups according to NFL usage, and nutritional intake was analyzed by the 24-hour recall method. Complex-sample multiple logistic regression analysis was used to determine the odds ratios (ORs) and 95% confidence intervals (CIs) for meeting the EARs according to NFL usage in the crude and adjusted (including metabolic conditions) models. The primary finding was that awareness and NFL use were associated with adequate intake above the EARs for vitamin A, vitamin B_2_, niacin, folate, Ca, Fe, and P; this association was more evident during the pandemic. After adjusting for covariates, during the pandemic, the ORs of meeting the EARs in the Aware+noUse group and Aware+Use group were 1.25 (CI 1.06–1.47) and 1.36 (CI 1.05–1.75) for vitamin A, 1.26 (CI 1.06–1.50) and 1.54 (CI 1.19–2.00) for vitamin B_2_, 1.32 (CI 1.13–1.56) and 1.46 (CI 1.15–1.85) for folate, and 1.46 (CI 1.06–2.00) and 1.73 (1.09–2.75) for P, respectively. Additionally, the ORs for niacin (1.21, 1.02–1.43) and Fe (1.29, 1.08–1.54) were significant in the Aware+noUse group, and that for Ca (1.39, 1.08–1.78) was significant in the Aware+Use group (all *p* <0.05). In conclusion, NFL awareness and use are associated with meeting the EARs for vitamins and minerals not listed on NFLs. For future recurring health crises, effective NFL use is necessary for healthy dietary practices.

## Introduction

Food labeling has an important role in helping consumers reach their health goals, making them consider the nutritional impact of their food choices [1,2]. Nutrition fact labels (NFLs), which are displayed on prepackaged foods, have been established as a one-size-fits-all public health tool in many countries worldwide for decades [3–5]. To date, the positive impact of NFLs on dietary intake has primarily involved energy and macronutrients [6,7]. Additionally, NFLs have been studied as a tool to avoid the consumption of nutrients that have negative impacts on health. For example, the role of NFLs in reducing the intake of beverages with added sugar [8], calories from saturated fats [9], and sodium [10] has been empirically revealed in previous studies. In addition to the impact of the nutrient information listed above, for which inclusion in NFLs is mandatory, it was also found that NFLs partially mediate attitudes toward overall healthy food choices. In particular, increased consumption of fresh fruits and vegetables and increased fiber intake were associated with frequent NFL reading [11]. The coronavirus disease 2019 (COVID-19) pandemic marked the beginning of 2020, and the spread of the disease has affected public health worldwide. As a result of the pandemic situation, face-to-face health and nutrition consultations with experts were impossible [12,13], and the reliability of nutritional information on unauthorized websites is reported to be very low [14]. Thus, this study hypothesized that the credibility of NFLs increased further due to the health crisis of the pandemic, and this trend may have led to healthier nutritional choices.

Due to the pandemic, many special nutritional modifications have been proposed; health professionals generally suggest eating a balanced diet with sufficient nutrients, such as foods rich in vitamins A, C, E, and B_6_, zinc, and iron (Fe), which are recommended for individual health [15]. Moreover, the government’s efforts to set appropriate intake levels and dietary guidelines for the public also continued [16,17]. Estimated average requirement (EAR) cutoff points were set to suggest intakes suitable for individuals in specific gender groups or life stages [18,19]. EARs are indicators that belong to the Dietary Reference Intakes (DRIs, also called Recommended Dietary Allowances (RDAs)) and represent the average daily nutrient level estimated to meet the requirements of 50% of healthy individuals. In particular, EARs are used to determine the proportion of the population that meets the EAR when assessing adequate intake [20]. Thus, meeting EARs has many public health implications; however, to date, research on the relationship between NFL use and meeting EARs, which are the government’s two major health concerns, and the association between the two, which would have been more evident during the pandemic, is insufficient.

The aim of this cross-sectional study was to evaluate the odds ratio between NFL use and meeting the EARs for vitamins and minerals that are not needed to be included on labels among Korean adults (aged 19 to 85 years old) using state-of-art Korea National Health and Nutrition Examination Survey (KNHANES) VIII data covering both the prepandemic (2019, *n* = 6606) and pandemic periods (2020 and 2021, *n* = 12085). Subjects were included in the unAware+noUse, Aware+noUse, and Aware+Use groups according to NFL usage, and nutritional intake was analyzed by the 24-hour recall method to determine whether the EARs were met.

## Materials and methods

### Data collection and study participants of the KNHANES

In this study, data from the eight periods of the KNHANES (2019–2021) were used, and the total number of subjects for the 3 years was 22559. Among them, the number of subjects meeting the inclusion criteria stated below for the 3 years was 18691. In Korea, the first COVID-19 patient was diagnosed in early 2020, and pandemic waves occurred until the fall of 2021 [21]; therefore, 2020 and 2021 were set as the pandemic years in this study. The KNHANES is an annual nationally representative survey based on official government statistics (Article 16 of the National Health Promotion Act, Statistics approval number 117002). Over the past 20 years, this survey has been considered to provide accurate, timely, and nationwide health statistics, including health interview, health examination, and nutritional survey results [22]. The KNHANES is conducted by the Korea Disease Control and Prevention Agency (KDCA) as instructed by the Ministry of Health and Welfare of Korea. The KNHANES uses Korea’s population and housing census data as a sampling frame, and the survey is conducted in respondents’ homes by professionally trained interviewers using a computer-assisted personal interviewing (CAPI) method. The sample is drawn by multilayer stratification, and weights are provided for complex sample analysis. For the development of internal and external quality, public‒private partnerships among 180 experts have been formed by academic societies, and improvements in mobile examination centers (MEC) have been introduced. The annually collected KNHANES data from 1995 are publicly available on the KDCA web page (https://knhanes.kdca.go.kr).

## Inclusion criteria and the NFL group

The inclusion criterion was Korean adults aged 19 to 85 years. Because nursing homes and hospitals are excluded from the survey districts, all included subjects stayed in local residences and were not in an acute disease state. This study established inclusion groups according to the following criteria. 1) Excluding the population in the growth stage of infants, children, and adolescents (excluding the population under 19 years of age), 2) for the representativeness of this study, an age cutoff that can represent more than 80% of the entire Korean population was used; the population distribution of 19–85 years old, which accounts for 82.40% of the total Korean population, was selected. Rather, it accounts for 74.53% of the total population for those aged 19–75 and 65.57% for those aged 19–65. Additionally, the age category of 19–85 years includes 97.67% of the entire Korean adult (19 or over) population (Korean Statistical Information Service and National Statistics Portal, as of November 2023).

**Fig 1:**
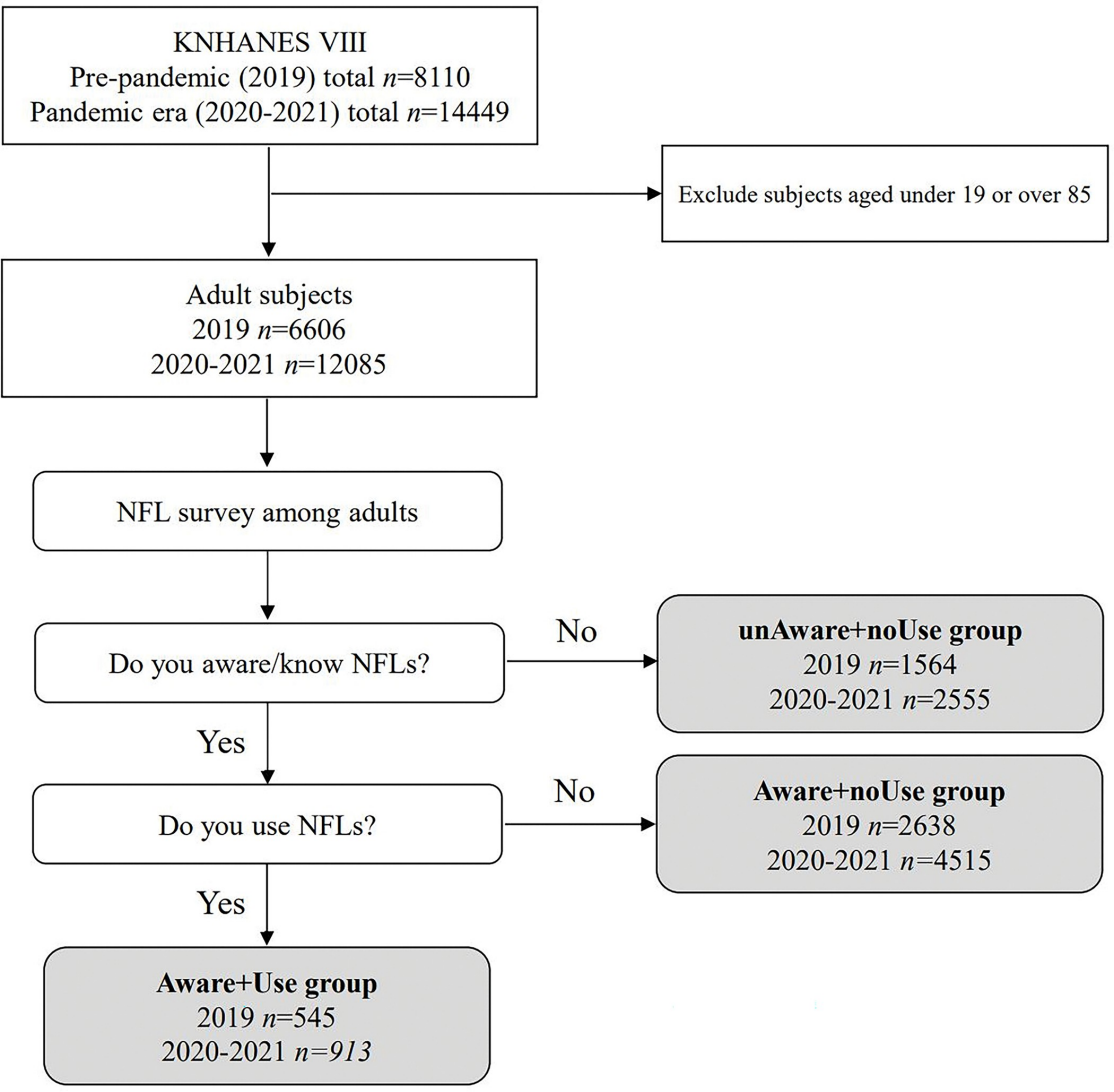
Flow diagram of the study group.

There may be the following potential biases in setting these age criteria. 1) Given that the elderly group aged 65–85 years was included, it is possible that diet and nutrient intake were affected by aging-related diseases. 2) As the proportion of young participants in the overall study group was relatively small (compared to previous studies that mainly studied people aged 19–65 years old), there is a possibility that the likelihood of study subjects consuming sufficient nutrients may be underreported. Therefore, this study attempted to minimize bias due to inclusion criteria in the analysis of the relationship between NFL use and vitamin and mineral intake by using stratified weights provided by KNHANES and correcting the following variables as covariates: age and sex, metabolic conditions, energy and macronutrient intake, etc. (more detail in each section below). After including only 19- to 85-year-old subjects, the questions used to divide participants into groups according to NFL usage were as follows: Are you aware of/do you know about NFLs? Do you use NFLs? **Fig 1** shows the flow diagram of the study group.

### Ethical review

The KNHANES was approved by the Institutional Review Board of the KDCA (2018-01-03-C-A) and conducted in accordance with the tenets of the Declaration of Helsinki. Written informed consent was obtained from all subjects before survey participation. All data were deidentified for research purposes, and the analysis was conducted in accordance with the guidelines and regulations of the KCDA.

### Household surveys, health behavior interviews, and health checkup examinations

The KNHANES health survey consists of three major parts: household surveys, health behavior interviews, and health checkup examinations. In the household survey, data on sex, age, the number of family members, the type of housing and residence, level of education, and household/individual income were collected. To evaluate health status and health behavior, information on disease history and/or current diseases, physical activity, daily life restrictions due to physical problems, working hours, medical facility use, vaccination status, subjective health status, quality of life, smoking status, alcohol consumption (frequency and amount), depression and stress, safety awareness, and pregnancy was obtained. As part of the health behavior investigation, information related to nutrition and dietary intake was also collected, detailed in a separate section below. Medical checkup examinations and anthropometric measurements were conducted; data on height, weight, waist circumference, blood pressure and pulse and blood (hematology and biochemistry) and urinary test results were collected. From the above measurement data, this study calculated body mass index (BMI = weight (kg)/height (m^2^), [23]), and the number of metabolic syndrome risk factors was counted [24]. The following metabolic syndrome criteria were taken from the National Cholesterol Education Program (NCEP) Adult Treatment Panel III (ATP III) guidelines: a high blood pressure (≥130/85 mmHg) or treatment for hypertension (HTN); a fasting plasma glucose level ≥100 mg/dL or treatment for diabetes mellitus (DM); a fasting plasma triglyceride (TG) level ≥150 mg/dL; a high-density lipoprotein (HDL)-cholesterol level <40 or <50 mg/dL in males and females, respectively; and a waist circumference >90 or 80 cm (Asian-Pacific criteria) in females and males, respectively.

### Dietary intake analysis with a 24-hour recall survey

**Fig 2:**
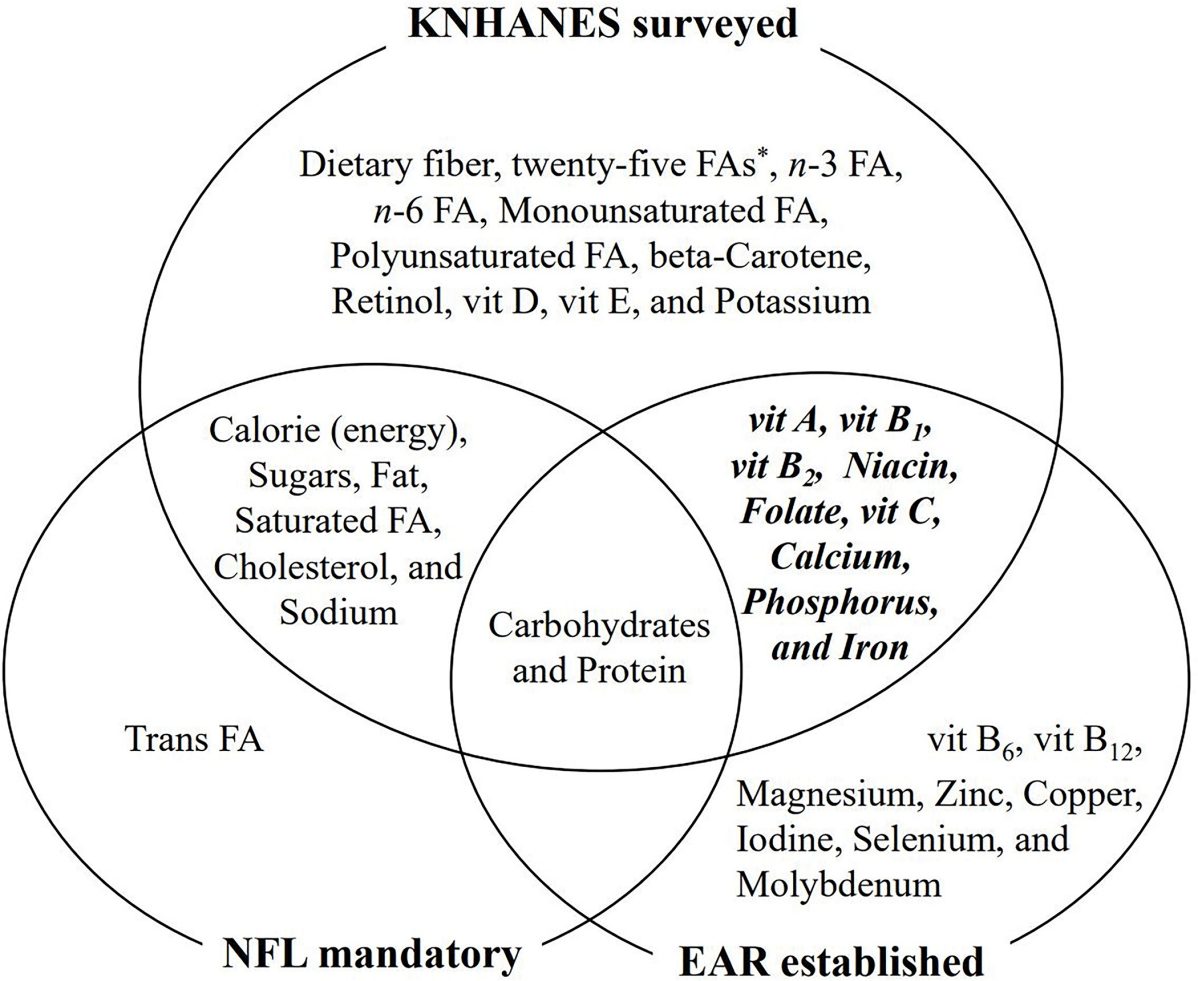
Venn diagram of the analyzed nutrients in the KNHANES, nutrients needed to be included in NFLs, and established nutrient EARs. Vit; vitamin, FAs; fatty acids, *Capric acid, Lauric acid, Myristic acid, Palmitic acid, Stearic acid, Arachidic acid, Behenic acid, Lignoceric acid, Myristoleic acid, Palmitoleic acid, Oleic acid, Eicosenoic acid, Erucic acid, Nervonic acid, Linoleic acid, Alpha-linolenic acid Gamma-linolenic acid, Stearidonic acid, Eicosadienoic acid, Dihomogamma-linolenic acid, Arachidonic acid, Eicosapentaenoic acid, Docosadienoic acid, Dotopentaenoic acid, Docohexaenoic acid.

A single 24-hour recall (one day before the survey) was used for the analysis of each individual’s dietary intake. Data on the following aspects were collected: water intake, food name, food code and name, total amount of food cooked (volume, weight), food intake (volume, weight), product name, manufacturer name, food group classification, and intake amount. Based on the Food Ingredient Table database (Rural Development Administration of Korea, 2020, ver. 9.2), food intake was converted into nutrient data. By adding the intakes of all nutrients in a day, daily intake data for 52 nutrients were calculated (**Fig 2**).

### Nutrients needed to be included in NFLs and established EARs for nutrients in Korea

**Fig 2** shows the nutrients needed to be included in NFLs and the established EARs for nutrients in Korea, presented in a Venn diagram format. In Korea, NFLs were introduced in 1994, concurrent with the enforcement of the US Nutrition Labeling and Education Act of 1994 [25]. Currently, in Korea, NFLs must include the amount of calories, sodium, carbohydrates, sugars, fat, trans fat, saturated fat, cholesterol, and protein, the percentage of the product’s nutrients in the total daily value (DV%) and the serving size portion [26]. Regarding DRIs, with the enactment of the National Nutrition Management Act in 2010, the management of all work related to DRIs for Koreans shifted from the private sector and academia to the government [27]. Among the various standards of DRIs, EARs are values calculated from the median daily nutrient requirements of Koreans, which means that the EARs can satisfy the needs of 50% of the population [28,29]. EARs can be set if there is a sufficient scientific basis for the needed amount of nutrients and calculated from the median daily nutrient requirements of healthy people [30]. Additionally, EARs are mainly used to assess Korea’s aims to reduce the frequency/proportion of the population eating less than the EARs from a public health standpoint. In Korea’s DRIs, the EARs for 19 nutrients have been established. Among the nutrients surveyed in the KNHANES, vitamins/minerals not listed on NFLs and for which EARs have been established include vitamin (vit) A, vitamin B_1_, vitamin B_2_, niacin, folate, vitamin C, calcium, phosphorus (P), and Fe. The EARs for the above nine nutrients are shown in **Table 1.**

**Table 1:** Estimated average requirements (EARs).

/day	vit A (μg RAE)		vit B_1_ (mg)		vit B_2_ (mg)		Niacin (NE mg)		Folate (μg DFE)	vit C (mg)	Calcium (mg)		Phosphorus (mg)	Fe (mg)	
Age	M	F	M	F	M	F	M	F	M/F	M/F	M	F	M/F	M	F
19–29	570	460	1.0	0.9	1.3	1.0	12	11	320	75	650	550	580	8	11
30˗49	560	430													
50–64	530	410									600				6
65–74	510		0.9	0.8	1.2	0.9	11	10						7	
> 75	500			0.7	1.1	0.8	10	9							5

Daily reference intake for Koreans (issued by Ministry of Health and Welfare and Korean Nutrition Society, 2020). M; male, F; female, Vit; vitamin, RAEs; retinol activity equivalents, NEs; niacin equivalents, and DFEs; dietary folate equivalents.

### Multiyear, complex-sample weighted analysis and statistical methods

To integrate the prepandemic (2019) and pandemic period complex samples (2020–2021), the appropriate strata, layer, and colony weights were applied. General characteristics are presented as numerical values and percentages (categorial variables) and means ± standard errors (SEs, noncategorical variables). Average estimations of daily energy, macronutrient (energy intake adjusted), and vitamin/mineral intake (energy and macronutrient adjusted) according to NFL use were conducted by a general linear model. The proportion of subjects who met the EARs according to NFL use was analyzed in both the prepandemic and pandemic periods by crosstabulation (weighted percentages (%) and unweighted frequencies for the number of subjects). Complex-sample multiple logistic regression analysis was used to determine the odds ratios (ORs) and 95% confidence intervals (CIs) for meeting the EARs (adequate intake) according to NFL usage in both the crude model and the adjusted model. Sex, age, region of residence, income quartile, education level, and energy, carbohydrate, protein, and fat intake were included as covariates. In addition, since the chronic disease-related health status (which may affect dietary intake and NFL use) of the subjects was not considered in the inclusion criteria, the number of risk factors for metabolic syndrome was also included as a covariate. All data were analyzed using SPSS version 26.0 (IBM/SPSS Corp., Chicago, IL, USA), and a two-tailed *P* value of less than 0.05 was considered statistically significant.

## Results

### Basic characteristics of the participants

**Table 2** outlines the basic characteristics and NFL use of the adult participants of the 2019 and 2020–2021 KNHANES. In the prepandemic and pandemic periods, the average age of the subjects was 48.1±0.5 years old and 48.6±0.4 years old, respectively, and male subjects accounted for 44.7% and 44.6% of the sample, respectively. Among the metabolic conditions, there were significant differences in the proportion of participants with DM, low HDL levels, and high TG levels between the two periods (p<0.05), and 78.4% and 80.1% of the subjects lived in urban areas (p = 0.005). A total of 36.2% to 37.6% of the subjects had a college degree or higher education in the prepandemic and pandemic periods. Except for subjects who did not respond to the NFL survey, NFL use information was collected from the subjects, and there was no difference by period.

**Table 2:** Basic characteristics of the study participants.

	Prepandemic 2019 (n = 6606)	Pandemic-era 2020–2021 (n = 12085)	*P*
Age, mean and n (%)	48.1±0.5	48.6±0.4	0.371
19-49y (young)	2680 (40.6)	5089 (42.1)	
50-85y (middle age and elderly)	3926 (59.4)	6996 (57.9)	
Sex, n (%)			0.417
Male	2956 (44.7)	5387 (44.6)	
Female	3650 (55.3)	6698 (55.4)	
BMI, mean (kg/m^2^)	23.9±3.6	24.1±3.7	0.058
Region of residence, n (%)			0.005
Urban area	5293 (80.1)	9472 (78.4)	
Rural area	1313 (19.9)	2613 (21.6)	
Education level, n (%)			0.402
Middle school or below	1649 (30.2)	3001 (32.3)	
High school	1980 (33.4)	3689 (34.4)	
College or over	2293 (36.2)	4036 (37.6)	
NFL use status, n (%)^a^			0.513
unAware+noUse	1564 (23.7)	2555 (21.1)	
Aware+noUse	2638 (39.9)	4515 (37.4)	
Aware+Use	545 (8.3)	913 (7.6)	
Metabolic conditions, n (%)^b^			
DM	2440 (36.9)	4796 (39.7)	<0.001
HTN	2720 (41.2)	4990 (41.3)	0.445
Abdominal obesity by WC	2880 (43.6)	5532 (45.8)	0.196
Low HDL cholesterol	1709 (25.9)	3453 (28.6)	0.014
High TG	1741 (26.4)	3026 (25.0)	0.025

Mean±standard error (SE) or the number of subjects. *P* values were derived from Pearson tests (categorical variables) and ANOVA tests (continuous variables). *P* < 0.05 was considered significant. ^a^Except for subjects who refused to answer, did not know, and had no response. ^b^Metabolic conditions criteria were from the National Cholesterol Education Program (NCEP) Adult Treatment Panel III (ATP III) guidelines. BMI; body mass index. DM; diabetes mellitus, HTN; hypertension, WC; waist circumference, HDL; high-density lipoprotein cholesterol, TG; triglyceride.

### Daily energy and nutrient intake and the proportion of subjects who met the EARs according to NFL use

**Table 3** shows the population-weighted daily average energy and nutrient intakes and the proportion of subjects who met the EARs according to NFL use in the prepandemic and pandemic periods. The average intake of energy varied from 1745.5±29.5 kcal/d to 2046.7±24.3 kcal/d in the prepandemic period and from 1710.8±26.2 kcal/d to 1956.8±19.2 kcal/d in the pandemic period. After energy intake was included as a covariate, daily average carbohydrate, protein, and fat intake was calculated for each NFL group. Additionally, daily vitamin A, vitamin B_1_, vitamin B_2_, niacin, folate, vitamin C, calcium, phosphorus, and Fe intake were evaluated after including macronutrients as covariates (statistically not significant among the three groups in each period). The proportion of subjects who met the EARs and the number of subjects in each NFL group in the pre- and pandemic periods are also shown in **Table 3.** In the pandemic period, the lowest proportion of subjects meeting the EAR for vitamin A ranged from 19.8% to 25.4% by NFL use, with the largest proportion meeting the EAR for P ranging from 77.2 to 84.4% by NFL use. The significant differences in the proportion of subjects meeting each nutrient’s EAR among the unAware+noUse, Aware+noUse, and Aware+Use groups were analyzed by using a crude model, as described in the next section.

**Table 3:** Daily energy and nutrient intake and the proportion of subjects who met the EARs according to NFL use in the pre- and pandemic periods.

		Prepandemic			Pandemic era	
	unAware+noUse (n = 1564)	Aware+noUse (n = 2638)	Aware+Use (n = 545)	unAware+noUse (n = 2555)	Aware+noUse (n = 4515)	Aware+Use (n = 913)
Energy (kcal)	1745.5±29.5	2046.7±24.3	1869.9±48.0	1710.8±26.2	1956.8±19.2	1826.9±31.5
Carbohydrates^†^ (g)	289.9±2.6	280.7±1.9	255.0±3.9	277.8±2.6	268.2±1.4	251.4±2.7
Protein^†^ (g)	68.8±0.7	72.5±0.6	76.2±1.3	67.9±0.7	71.1±0.4	74.3±0.9
Fat^†^ (g)	41.1±0.8	47.5±0.6	56.1±1.3	42.7±0.7	48.4±0.9	54.5±0.9
vit A (μg RAE)^‡^	380.6±15.4	376.6±8.8	362.4±13.0	361.4±8.1	382.3±6.8	398.7±11.6
≥EAR, n (%)	275 (18.8)	632 (25.2)	121 (21.7)	467 (19.8)	1104 (24.8)	232 (25.4)
vit B_1_ (mg)^‡^	1.4±0.0	1.3±0.0	1.3±0.0	1.2±0.0	1.2±0.0	1.1±0.0
≥EAR, n (%)	995 (67.0)	1751 (71.6)	309 (60.1)	1307 (55.9)	2615 (61.9)	470 (54.9)
vit B_2_ (mg)^‡^	1.5±0.0	1.6±0.0	1.7±0.0	1.6±0.0	1.6±0.0	1.6±0.0
≥EAR, n (%)	770 (54.3)	1737 (70.7)	371 (71.7)	1329 (57.1)	2980 (70.6)	635 (73.1)
Niacin (NE mg)^‡^	12.9±0.2	12.8±0.1	13.2±0.3	12.4±0.1	12.7±0.1	12.3±0.2
≥EAR, n (%)	563 (28.7)	1281 (35.8)	247 (37.7)	874 (38.6)	2051 (49.9)	375 (44.5)
Folate (μg DFE)^‡^	324.4±5.2	315.1±3.4	317.5±7.9	317.3±4.2	305.9±2.4	305.1±5.4
≥EAR, n (%)	574 (40.1)	1113 (44.1)	198 (35.8)	933 (39.4)	1846 (41.9)	297 (33.8)
vit C (mg)^‡^	61.7±3.5	63.5±1.6	72.7±3.9	61.7±3.3	66.8±2.3	66.8±3.6
≥EAR, n (%)	356 (23.0)	714 (28.1)	155 (29.1)	527 (22.5)	1197 (26.5)	214 (24.1)
Ca (mg)^‡^	496.1±8.0	498.9±6.2	513.5±12.7	483.7±7.5	485.1±4.7	500.1±9.4
≥EAR, n (%)	347 (25.2)	777 (31.6)	160 (30.4)	578 (25.1)	1286 (28.7)	251 (28.0)
P (mg)^‡^	1044.1±6.0	1045.6±4.4	1049.1±8.6	1045.5±6.3	1036.2±3.6	1039.0±7.3
≥EAR, n (%)	1092 (75.2)	2139 (87.5)	433 (84.7)	1844 (77.2)	3696 (87.0)	731 (84.4)
Fe (mg)^‡^	12.1±0.2	11.7±0.1	11.5±0.3	10.6±0.2	10.4±0.1	10.3±0.3
≥EAR, n (%)	1082 (72.7)	1757 (70.0)	287 (56.6)	1499 (61.3)	2687 (61.4)	415 (48.7)

Values are the mean±standard error (SE). ^†^Energy intake was used as a covariate, ^‡^ Energy, carbohydrate, protein, and fat intake were used as covariates for determining the weighted mean intake for each year by weighted complex sample general linear model analysis. ≥EAR; the number and proportion of subjects who met the estimated average requirement (EAR) for each nutrient, Vit; vitamin, RAEs; retinol activity equivalents, NEs; niacin equivalents, DFEs; dietary folate equivalents, Ca; calcium, P; phosphorus, Fe; iron.

### Association between NFL use and meeting EARs

The OR analysis of meeting EARs according to NFL use is presented in **Table 4**. Overall, the relation between NFL use and meeting EARs during the pandemic period was greater than that during the prepandemic period, and statistically significant cases are indicated in bold text in the model including basic information and adjusting for metabolic conditions (reference; unAware+noUse group in each period = 1.0). In the adjusted model for the prepandemic period, the OR of meeting the EAR for folate was 1.24 (95% CI: 1.02–1.52, p = 0.035) for the Aware+noUse group and 1.46 (1.04–1.51, p = 0.030) for the Aware+Use group. The OR of meeting the EAR for vitamin C was 1.23 (95% CI: 1.01–1.51, p = 0.045) for the Aware+noUse group and 1.57 (95% CI: 1.17–2.13, p = 0.003) for the Aware+Use group. The OR of meeting the EAR for P was 1.54 (95% CI: 1.10–2.18, p = 0.013) for the Aware+noUse group and 2.31 (95% CI: 1.26–4.23, p = 0.007) for the Aware+Use group.

**Table 4:** Odds ratios of meeting the EARs according to NFL use in the pre- and pandemic periods.

		Prepandemic				Pandemic era			
		Crude	*P*	Adjusted^†^	*P*	Crude	*P*	Adjusted^†^	*P*
Refer.	unAware+noUse	1.00							
vit A	Aware+noUse	1.45 (1.19–1.76)	0.000	1.13 (0.88–1.46)	0.343	1.34 (1.15–1.59)	0.000	**1.25** (1.06–1.47)	**0.009**
	Aware+Use	1.19 (0.92–1.55)	0.023	0.92 (0.65–1.30)	0.631	1.38 (1.11–1.71)	0.004	**1.36** (1.05–1.75)	**0.019**
vit B_1_	Aware+noUse	1.24 (1.03–1.50)	0.027	0.97 (0.73–1.27)	0.803	1.29 (1.12–1.47)	0.000	0.95 (0.80–1.13)	0.564
	Aware+Use	0.74 (0.59–0.93)	0.023	0.82 (0.59–1.14)	0.238	0.96 (0.79–1.17)	0.708	0.82 (0.62–1.08)	0.158
vit B_2_	Aware+noUse	2.03 (1.71–2.40)	0.000	1.14 (0.89–1.45)	0.300	1.80 (1.57–2.07)	0.000	**1.26** (1.06–1.50)	**0.010**
	Aware+Use	2.13 (1.62–2.80)	0.000	1.24 (0.85–1.81)	0.261	2.04 (1.05–1.50)	0.000	**1.54** (1.19–2.00)	**0.001**
Niacin	Aware+noUse	1.58 (1.33–1.88)	0.000	1.02 (0.80–1.30)	0.893	1.58 (1.39–1.80)	0.000	**1.21** (1.02–1.43)	**0.026**
	Aware+Use	1.38 (1.07–1.78)	0.013	1.28 (0.88–1.86)	0.195	1.27 (1.06–1.53)	0.010	1.17 (0.90–1.52)	0.231
Folate	Aware+noUse	1.18 (1.01–1.38)	0.023	**1.24** (1.02–1.52)	**0.035**	1.11 (0.97–1.27)	0.139	**1.32** (1.13–1.56)	**0.001**
	Aware+Use	0.83 (0.65–1.06)	0.132	**1.46** (1.04–2.04)	**0.030**	0.79 (0.65–0.94)	0.009	**1.46** (1.15–1.85)	**0.002**
vit C	Aware+noUse	1.31 (1.10–1.55)	0.018	**1.23** (1.01–1.51)	**0.045**	1.24 (1.07–1.44)	0.004	**1.24** (1.05–1.47)	**0.013**
	Aware+Use	1.38 (1.06–1.79)	0.002	**1.57** (1.17–2.13)	**0.003**	1.10 (0.87–1.37)	0.434	1.24 (0.96–1.60)	0.106
Ca	Aware+noUse	1.37 (1.17–1.62)	0.000	**1.21** (1.01–1.46)	**0.044**	1.20 (1.03–1.40)	0.017	1.16 (0.97–1.39)	0.112
	Aware+Use	1.30 (0.98–1.72)	0.023	1.39 (1.00–1.93)	0.053	1.16 (0.95–1.42)	0.143	**1.39** (1.08–1.78)	**0.011**
P	Aware+noUse	2.31 (1.92–2.78)	0.000	**1.54** (1.10–2.18)	**0.013**	1.98 (1.70–2.30)	0.000	**1.46** (1.06–2.00)	**0.020**
	Aware+Use	1.83 (1.052–1.650)	0.000	**2.31** (1.26–4.23)	**0.007**	1.60 (1.27–2.01)	0.000	**1.73** (1.09–2.75)	**0.021**
Fe	Aware+noUse	0.87 (0.75–1.02)	0.023	1.10 (0.88–1.37)	0.397	1.01 (0.88–1.15)	0.928	**1.29** (1.08–1.54)	**0.006**
	Aware+Use	0.49 (0.38–0.62)	0.023	1.11 (0.75–1.64)	0.608	0.60 (0.50–0.72)	0.023	1.11 (0.85–1.46)	0.441

Values are odds ratios (ORs) and 95% confidence intervals (CIs). *P* values were derived from the complex-sample multiple logistic regression analysis. *P* < 0.05 was considered significant, and significant ORs are shown in bold. ^†^Adjusted model; sex, age, region of residence, income quartile, education level, number of metabolic syndrome criteria met, energy, carbohydrates, protein, and fat intake were included as covariates. Refer.; the unAware+noUse group was used as a reference.

In the adjusted model for the pandemic period, the OR of meeting the EAR for vitamin A was 1.25 (1.06–1.47, p = 0.009) for the Aware+noUse group and 1.36 (95% CI: 1.05–1.75, p = 0.019) for the Aware+Use group; that of meeting the EAR for vitamin B_2_ was 1.26 (95% CI: 1.06–1.50, p = 0.010) for the Aware+noUse group and 1.54 (95% CI: 1.19–2.00, p = 0.001) for the Aware+Use group; that of meeting the EAR for folate was 1.32 (95% CI: 1.13–1.56, p = 0.001) for the Aware+noUse group and 1.46 (95% CI: 1.15–1.85, p = 0.002) for the Aware+Use group; and that of meeting the EAR for P was 1.46 (95% CI: 1.06–2.00, p = 0.020) for the Aware+noUse group and 1.73 (95% CI: 1.09–2.75, p = 0.021) for the Aware+Use group. In the adjusted model for the pandemic period, the following nutrients showed significant ORs in only one group (compared to the reference group): niacin, with an OR of 1.21 (95% CI: 1.02–1.43, p = 0.026) in the Aware+noUse group; vitamin C, with an OR of 1.24 (95% CI: 1.05–1.47, p = 0.013) in the Aware+noUse group; Ca, with an OR of 1.39 (95% CI: 1.08–1.78, p = 0.011) in the Aware+Use group; and Fe, with an OR of 1.32 (95% CI: 1.13–1.56, p = 0.001) in the Aware+noUse group.

## Discussion

Establishing the relationship between NFL use and dietary intake is important. This study of nationally representative survey data in Korea newly found that 1) the awareness and use of NFLs were associated with adequate intake above the EARs for vitamin A, vitamin B_2_, niacin, folate, Ca, Fe, and P, for which inclusion on NFLs is not needed, and 2) this association has become stronger during the pandemic than prepandemic periods.

The novelty of this is as follows: 1) it evaluated the relationship between adequate intake of nutrients not listed in the NFL and the use of the NFL; rather, previous studies were simply limited to the effectiveness of the NFL on macronutrients and energy intake listed in the NFL [7,9,31]; 2) the current study obtained nationally representative results using a stratified complex sample of 18691 people collected over 3 years and included a broad range of adults, from 19 to 85 years of age, for both men and women, whereas previous studies were limited to specific age groups [1], race groups [8], and disease statuses [6]; and 3) this study calculated the actual daily nutrient intake of the study subjects using the 24-hour recall method to confirm whether participants were consuming enough vitamins and minerals over the recommended amount; on the other hand, other studies just looked at healthy eating through indices [32,33] and simply surveyed the increased frequency of vegetable/fruit/fish intake [11,31,32].

NFLs have a broad and deep practical impact on food intake and appropriate nutrient intake [34]. Across countries, NFLs contribute to the improvement of public health by providing nutritional information for products to help consumers identify and choose the foods necessary for a healthy diet [35]. In previous limited studies, the health implications of NFLs were reported to be directly reducing energy and fat intake. Neuhauser et al. [31], using a telephone survey of 1435 residents, revealed that NFL use was significantly associated with lower fat intake. In their study, after controlling for all demographic, psychosocial, and behavioral variables, frequent NFL use explained 6% of the variance in fat intake. Another study also reported that the NFL user group showed significant differences in mean nutrient intakes with lower total energy, total fat, saturated fat, cholesterol, sodium, and sugar intake compared to non-NFL users [7]; however, that study was limited in that it was the result of not adjusting the basic characteristic with covariates.

In this study, during 2019–2022, the average NFL awareness ratio was approximately 46.6%, and the average usage ratio was low, at approximately 7.9%. These prevalences were lower than those reported in studies conducted in the US (75%, [36]), Canada (52%, [37]), and Europe (47%, [38]) targeting the general nationally representative population in each country. This is probably because the definitions of label ‘use’ varied among the studies. In this study, subjects were asked whether they used NFL information from the perspective of whether NFLs influenced their food selection (consideration); rather, in other countries, use probably meant reading or being aware of the labels. Additionally, there was no separate time frame in the KNHANES questionnaire, so when subjects reported using NFLs ‘always or often’, the answer would have been yes; however, previous studies had a limited period and asked whether subjects had NFL usage experience within 1 month [39] or 12 months [40]. Furthermore, the broader population of subjects up to 85 years of age included in this study, compared to that of adults up to 65 years old in other studies, may also have been related to the low NFL use rate.

To the best of the author’s knowledge, the present study is the first attempt to evaluate the relationship between NFL use and adequate micronutrient intake based on EARs for energy, carbohydrates, fat, and protein that were included as potential covariates in logistic regression models. Additionally, this study considered metabolic conditions as a covariate when calculating ORs. This is because, in previous studies, greater NFL use was reported in situations requiring dietary modifications and in subjects with different disease diagnosis statuses [41–43]. Ruopeng An analyzed 2005–2010 NHANES data, and the results showed that US adults diagnosed with diabetes/prediabetes were substantially more likely to use NFLs than those not diagnosed with diabetes [44]. Additionally, another study showed that hypertension patients had 71% higher odds of frequently using NFLs for sodium information than those without hypertension [45].

Therefore, why is higher use of NFLs related to meeting the EARs for vitamins and minerals not included in NFLs, why do subjects consume more vitamins and minerals than the EARs just by being aware of NFLs (Aware+noUse), and why was this association more pronounced in the pandemic period? The following are theoretical possibilities regarding the results of this study.

First, NFLs themselves have helped consumers make healthy food decisions for high vitamin and mineral intake. A study conducted in Spain measured the relative Mediterranean Diet score (rMED) and found that NFL users had higher adherence to a Mediterranean diet [32], yet that study did not examine the quantitative intake of individual nutrients. In that study, the NFL user group showed higher intake of fish (OR per 100 g/day: 1.94; 95 CI: 1.38–2.71), vegetables (OR per 100 g/day: 1.15; CI 95%: 1.08–1.12), and fruits (OR per 100 g/day: 1.22; 1.11–1.34). Additionally, according to a diet quality study using an income-stratified sample conducted in the US, the Healthy Eating Index (HEI, usually based on the ratios of the dietary constituents to energy [33]) was positively associated with both self-reported and objective measures of NFL use in univariate models. Thus, the connection between NFL use and MED/HEI scores, which are considered optimal in terms of adequate vitamin and mineral intake [46–48], supports the potential role of NFLs as a tool to meet EARs indicated in the results of this study.

Second, the use of NFLs may lead to interest in other health claims or additional information on food packaging. In Korea, in addition to conventional NFLs, food product providers/manufacturers can provide fortification labels and comparison labels for emphasis [49]. For example, high vitamin C [50], high Ca [51], or comparative phrases such as more/less can be labeled if the product is superior to others in the same product group. Often, it has been reported that people do not use NFLs even if they are aware of them due to difficulties in understanding the numeric/quantitative information in the table matrix format of traditional NFLs [52–54]. In these cases, the adjective labels [55] or minimal numerical content symbols [56] can be helpful, and alternatively, simple additional information near the NFL on the package may be helpful in making healthy food choices. Additionally, Kozup et al. reported that when favorable nutritional information or health claims are presented on products, consumers have more favorable attitudes toward their purchase decisions [57]. In addition, by conveying the nutritional characteristics of foods through explanatory information such as none, low, high, rich, and fortified, NFLs have become easier for consumers to use, and sales of claimed foods have also increased [58]. Additional claims, currently being managed with NFLs in Korea, would have helped the Aware+noUse group select foods that were rich in vitamins and minerals.

Third, the percent daily values (DVs) displayed in NFLs would have helped subjects select nutrient-dense foods instead of empty calorie foods, which would have helped them meet the EARs for vitamins and minerals. Inclusively, nutrient-dense foods and the nutritional quality of individual foods are emphasized for the consumption of more beneficial nutrients in the overall diet [59]. DVs are science-driven and user-friendly nutrient profiling systems that rate individual foods [60]. For example, the US Food and Drug Administration (FDA) indicates whether a food is “healthy” based on the DV, which is used if it provides ≥10% DV of protein, fiber, vitamin A, vitamin C, calcium, or Fe [61]. In particular, it was verified that using sums and means for nutrient DVs well explains the concept of the composite nutritional quality index related to nutrient-rich foods [62]. In this study, the probability of meeting the EAR in the Aware+Use group was higher than that in the Aware+noUse group, indicating that NFL content consideration (use) had a greater correlation with meeting the EAR. Additionally, as NFL ‘use’ means that food choices are influenced by NFL information in the KNHANES enquiry, it can be highly inferred that the Aware+Use group also uses DV information, and this would be effective and useful in helping them make more nutrient-rich food choices [63].

Fourth, NFLs probably gained credibility during the pandemic as the most prominent source of nutrition information. In the wake of the pandemic, information seeking and the practice of healthy eating notably increased [64,65]. Specifically, the “spread of rumors and massive information” were major obstacles to obtaining health information, and thus, “social media accounts of official health organizations” were ranked as the most relied upon information source during the pandemic [66]. In terms of reducing nutrition confusion, there may be increased reliance on obtaining nutritional information from official sources such as NFLs. New and unregulated forms of information have raised undue concerns or even been misunderstood [67,68]. Thus, NFLs, which are universally accessible and uniformly managed without the need for technology or devices, may have become a more credible and popular source of nutritional information. Furthermore, positive eating habit changes after the pandemic were noted in a study in Poland [69]. In that study, over 60% of all participants reported starting to eat more nutritious and regular meals after the COVID-19 pandemic. As such, the health crisis itself may have led to active health behaviors, which may have had a practical impact on adequate nutrient intake through NFL use.

Last, due to hygiene-related issues during the COVID-19 pandemic, the consumption of packaged foods such as ready meals and home meal replacements [70] increased compared to foods purchased from farmers markets [71]. Considering that most food packages have NFLs, frequent exposure to NFLs while purchasing packaged food during the COVID-19 pandemic may have helped consumers obtain appropriate nutritional information. Several studies reported that a better understanding of NFLs was associated with frequent label use [38,72]. There is additional evidence that a positive attitude and motivation to use labels could lead to better comprehension about the nutrition information provided in labels [73,74]. According to the results of research from the perspective of health behavior theories [75], perceived benefits, the perceived importance of nutrition in food shopping, and the perceived importance of a healthy diet led to searching for information on food labels, and this behavior is related to the consumption of a healthy diet. Given the potential explanation that the pandemic led to a higher desire to explore nutrition information and develop healthy behavior, the relationship between NFL use and meeting EARs may also be explained.

To further present Korea’s typical dietary intake patterns for readers’ additional information, the DRIs for Koreans present the ‘Energy adequacy ratio’ as the primary dietary intake recommendation as follows: the recommended ratio of carbohydrates to total energy is 55–65%, the recommended ratio of protein is 7–20%, and the recommended ratio of total fat is 15–30% (for both adult men and women). Next, the recommended daily calories for men are 2600–1900 kcal/day, and for women, they are 2000–1500 kcal/day (sequentially decreasing from age 19 to age 75 or older, [17]). According to the Korea Health Industry Development Institute (KHIDI), although it is a limited statistic that includes the entire Korean population older than 1, energy intake in 2019 and 2020 was 1871.79±16.5 kcal/day and 1914.69±15.67 kcal/day, respectively [76]. In addition, the average carbohydrate, protein, and fat intake of Korean citizens in 2020 was 266.53±1.91 g/day, 70.16± 0.81 g/day, and 49.00±0.80 g/day, respectively, and the energy and macronutrient intake of the subjects of this study showed the same range as the average results from KHIDI statistics and DRI. Moreover, although there are no studies that have published the percentage of Koreans meeting the EAR for vitamins and minerals, the daily average intake analysis of each nutrient consumed by all Korean populations (over 1 year old) is as follows. Vit A; 379.35±8.57μgRAE (below EAR), vit B_1_; 1.23±0.01 mg, vit B_2_; 1.68±0.02 mg, niacin; 12.37±0.17 mg, folate; 296.77±3.17μgDFE (below EAR), vit C; 63.82±1.72 mg (below EAR), Ca; 487.68±5.87 mg (below EAR), P 1020.87±9.54 mg, and Fe; 10.76±0.12 mg [76]. The average values of each vitamin and mineral in this study did not differ from the range of previous results (mean±SE).

A few limitations of the present study should be noted. First, to eliminate potential bias, this study designed an adjusted model including as many covariates as possible, but there is a risk of cumulative type Ⅰ error due to covariates that may still exist. For example, factors such as NFL education experience, level of basic nutritional knowledge, or consultation experience with a dietitian, which were not investigated in this study, may have influenced appropriate vitamin/mineral intake. Second, although this study has the advantage of being long-term and using a large group, the typical diet of East Asians, including Koreans, is different from the Western diet in terms of higher vegetable consumption [77], so caution is needed when generalizing the results of this study to other countries. Third, the EARs for nutrients were limited to a small number of vitamins and minerals, making it impossible to analyze the relationship between NFL use and the consumption of other nutrients for which individuals are known to have reduced intake [78–80]. Last, the current study had a cross-sectional design, and the conclusion of this study should not be interpreted as a causal relationship.

The current study uniformly applied the EAR announced during the prepandemic period, but there may be changes in the EARs and/or minimum intake amounts of vitamins and minerals due to the pandemic. Thus, it is necessary for future research to estimate the changed value of vitamins and minerals and determine whether adequate intake is met by the renewed references. Furthermore, as suggestion for next study, it would be better to compare NFL use with multiple public health claims in meeting EARs. For example, survey data on whether people know or utilize health rules, traffic light signs, front labels, etc. [81] are available at the national level, it would be valuable to compare the relevance of meeting EARs with other public health information.

This study offers some important implications that may directly benefit the public. First, the most important implication of this study is that public campaigns to increase awareness of NFLs could improve the nutritional intake status of NFL-not-listed vitamins and minerals of the population. In other words, it can be said that the general public’s use of or awareness of the NFL is more influential than what nutrient numerical information is included in the content. Second, rather than increasing the understanding of traditional NFLs, policymakers should put more effort into continuously increasing public ‘awareness’ of NFLs on food packages. This is because simply being aware of the NFL can increase the relevance of consuming sufficient vitamins and minerals. Third, information accompanied by symbols and rating presentations can have effectiveness for adequate intake [82,83]. Therefore, there is a need for innovative approaches to develop various and simple descriptors for identifying healthy food options so that the public can use and recognize NFL easily and quickly. Last, the use of NFLs on fruits, vegetables, and raw produce is optional, so if NFL is mandatorily extended to broader food categories, NFLs may have a greater impact on sufficient vitamin and mineral intake. Applying NFLs to more prepared foods or fresh foods and frequently providing food packaging information could help ensure sufficient nutritional intake beyond EARs.

## Conclusion

In conclusion, the findings from the present study of the KHANES VIII indicate that NFL awareness and use is associated with meeting the EARs for vitamins and minerals not listed on NFLs. Although NFLs alone are not expected to be sufficient to modify behavior and ultimately improve health outcomes, a great impact of NFLs on vitamin and mineral intake during a communicable disease pandemic was confirmed. For future recurring health crises, it is necessary to promote the effective use of NFLs to develop healthy dietary practices.

## References

[1] Feunekes GI, Gortemaker IA, Willems AA, Lion R, Van Den Kommer M. Front-of-pack nutrition labelling: testing effectiveness of different nutrition labelling formats front-of-pack in four European countries. Appetite. 2008;50(1):57–70. doi: 10.1016/j.appet.2007.05.009 17629351

[2] Capacci S, Mazzocchi M, Shankar B, Brambila Macias J, Verbeke W, Pérez-Cueto FJ, et al. Policies to promote healthy eating in Europe: a structured review of policies and their effectiveness. Nutrition reviews. 2012;70(3):188–200. doi: 10.1111/j.1753-4887.2011.00442.x 22364161

[3] Food U, Administration D. Guide to nutrition labeling and education act (NLEA) requirements. Silver Spring, MD: Division of Field Investigations, Office of Regional Operations, Office of Regulatory Affairs, US Food & Drug Administration. 1994.

[4] Go Canada. Regulations amending the food and drug regulations (nutrition labelling, nutrient content claims and health claims). The Canada Gazette, Part II. 2003;137:154–403.

[5] Przyrembel H. Food labelling legislation in the EU and consumers information. Trends in Food Science & Technology. 2004;15(7–8):360–5.

[6] Post RE, Mainous AG III, Diaz VA, Matheson EM, Everett CJ. Use of the nutrition facts label in chronic disease management: results from the National Health and Nutrition Examination Survey. Journal of the American Dietetic Association. 2010;110(4):628–32. doi: 10.1016/j.jada.2009.12.015 20338291

[7] Ollberding NJ, Wolf RL, Contento I. Food label use and its relation to dietary intake among US adults. Journal of the American Dietetic association. 2011;111(5):S47–S51. doi: 10.1016/j.jada.2011.03.009 21515135

[8] Kim EJ, Ellison B, McFadden B, Prescott MP. Consumers’ decisions to access or avoid added sugars information on the updated Nutrition Facts label. PloS one. 2021;16(3):e0249355. doi: 10.1371/journal.pone.0249355 33780506 PMC8007016

[9] Huth PJ, Fulgoni VL, Keast DR, Park K, Auestad N. Major food sources of calories, added sugars, and saturated fat and their contribution to essential nutrient intakes in the US diet: data from the National Health and Nutrition Examination Survey (2003–2006). Nutrition journal. 2013;12:1–10. 23927718 10.1186/1475-2891-12-116 PMC3751311

[10] Kim M, Lopetcharat K, Gerard P, Drake M. Consumer awareness of salt and sodium reduction and sodium labeling. Journal of food science. 2012;77(9):S307–S13. doi: 10.1111/j.1750-3841.2012.02843.x 22957915

[11] Graham DJ, Laska MN. Nutrition label use partially mediates the relationship between attitude toward healthy eating and overall dietary quality among college students. Journal of the Academy of Nutrition and Dietetics. 2012;112(3):414–8. doi: 10.1016/j.jada.2011.08.047 22896856 PMC3561724

[12] Lucini D, Gandolfi CE, Antonucci C, Cavagna A, Valzano E, Botta E, et al. # StayHomeStayFit: UNIMI’s approach to online healthy lifestyle promotion during the COVID-19 pandemic. Acta Bio Medica: Atenei Parmensis. 2020;91(3):e2020037. doi: 10.23750/abm.v91i3.10375 32921731 PMC7716948

[13] Brunton C, Arensberg MB, Drawert S, Badaracco C, Everett W, McCauley SM, editors. Perspectives of registered dietitian nutritionists on adoption of telehealth for nutrition care during the COVID-19 pandemic. Healthcare; 2021: MDPI. doi: 10.3390/healthcare9020235 33672179 PMC7926532

[14] Fernandez MA, Caretero A, Jacob E, Karatha J, Raine KD. 17 Credibility and reach of nutrition influencers on social media. BMJ Specialist Journals; 2022.

[15] Naja F, Hamadeh R. Nutrition amid the COVID-19 pandemic: a multi-level framework for action. European journal of clinical nutrition. 2020;74(8):1117–21. doi: 10.1038/s41430-020-0634-3 32313188 PMC7167535

[16] Morgan-Bathke M, McLimans K, Temple NJ. Trends in Dietary Recommendations: Nutrient Intakes, Dietary Guidelines, and Food Guides. Nutritional Health: Strategies for Disease Prevention: Springer; 2023. p. 249–60.

[17] Ministry of Health and Welfare TKNS. Dietary reference intakes for Koreans 2020. Sejong 2020.

[18] De Lauzon B, Volatier J, Martin A. A Monte Carlo simulation to validate the EAR cut-point method for assessing the prevalence of nutrient inadequacy at the population level. Public health nutrition. 2004;7(7):893–900. doi: 10.1079/phn2004616 15482615

[19] Intakes IoMSCotSEoDR. DRI Dietary Reference Intakes: applications in dietary assessment. 2000. 25057725

[20] Park J, Yeo Y, Yoo JH. Dietary intake and nutritional status in young and middle-aged adults according to the meal frequency from the Korea National Health and Nutritional Survey. Korean Journal of Family Medicine. 2022;43(5):319. doi: 10.4082/kjfm.21.0149 36168904 PMC9532190

[21] Seong H, Hyun HJ, Yun JG, Noh JY, Cheong HJ, Kim WJ, et al. Comparison of the second and third waves of the COVID-19 pandemic in South Korea: Importance of early public health intervention. International Journal of Infectious Diseases. 2021;104:742–5. doi: 10.1016/j.ijid.2021.02.004 33556610 PMC7863747

[22] Oh K, Kim Y, Kweon S, Kim S, Yun S, Park S, et al. Korea National Health and Nutrition Examination Survey, 20th anniversary: accomplishments and future directions. Epidemiology and health. 2021;43. doi: 10.4178/epih.e2021025 33872484 PMC8289475

[23] Nuttall FQ. Body mass index: obesity, BMI, and health: a critical review. Nutrition today. 2015;50(3):117. doi: 10.1097/NT.0000000000000092 27340299 PMC4890841

[24] Lipsy RJ. The National Cholesterol Education Program Adult Treatment Panel III guidelines. Journal of managed care pharmacy: JMCP. 2003;9(1 Suppl):2–5. doi: 10.18553/jmcp.2003.9.s1.2 14613351 PMC10437161

[25] Silverglade BA. The nutrition labeling and education act—progress to date and challenges for the future. Journal of Public Policy & Marketing. 1996;15(1):148–50.

[26] Kang H-N, Shin E-J, Kim H-N, Eom K-Y, Kwon K-I, Kim S-Y, et al. Food nutrition labeling (processing food, food service business) in Korea. Food Science and Industry. 2011;44(1):21–7.

[27] Shin S, Kim S, Joung H. Evidence-based approaches for establishing the 2015 Dietary Reference Intakes for Koreans. Nutrition Research and Practice. 2018;12(6):459–68. doi: 10.4162/nrp.2018.12.6.459 30515273 PMC6277316

[28] Yang E-J, Bang H-M. Nutritional status and health risks of low income elderly women in Gwangju area. The Korean Journal of Nutrition. 2008:65–76.

[29] Choi M-J, Park E-J, Jo H-J. Relationship of nutrient intakes and bone mineral density of elderly women in Daegu, Korea. Nutrition Research and Practice. 2007;1(4):328–34. 20368958 10.4162/nrp.2007.1.4.328 PMC2849042

[30] Paik HY. Dietary reference intakes for Koreans (KDRIs). Asia Pacific Journal of Clinical Nutrition. 2008;17. 18460441

[31] Neuhouser ML, Kristal AR, Patterson RE. Use of food nutrition labels is associated with lower fat intake. Journal of the American dietetic Association. 1999;99(1):45–53. doi: 10.1016/S0002-8223(99)00013-9 9917731

[32] Navarrete-Muñoz EM, Torres-Collado L, Valera-Gran D, Gonzalez-Palacios S, María Compañ-Gabucio L, Hernández-Sánchez S, et al. Nutrition labelling use and higher adherence to Mediterranean diet: results from the DiSA-UMH study. Nutrients. 2018;10(4):442. doi: 10.3390/nu10040442 29614009 PMC5946227

[33] Reedy J, Lerman JL, Krebs-Smith SM, Kirkpatrick SI, Pannucci TE, Wilson MM, et al. Evaluation of the healthy eating index-2015. Journal of the Academy of Nutrition and Dietetics. 2018;118(9):1622–33. doi: 10.1016/j.jand.2018.05.019 30146073 PMC6718954

[34] Campos S, Doxey J, Hammond D. Nutrition labels on pre-packaged foods: a systematic review. Public health nutrition. 2011;14(8):1496–506. doi: 10.1017/S1368980010003290 21241532

[35] Cowburn G, Stockley L. Consumer understanding and use of nutrition labelling: a systematic review. Public health nutrition. 2005;8(1):21–8. doi: 10.1079/phn2005666 15705241

[36] Statistics NCfH. Healthy People 2000: National Health Promotion and Disease Prevention Objectives: Healthy People 2000 Final Review: US Department of Health and Human Services, Centers for Disease Control and …; 2001.

[37] Ellis S. Consumer use and interpretation of trans fat information on food labels 2009. 10.3148/71.1.2010.6 20205970

[38] Drichoutis AC, Lazaridis P, Nayga RM, Kapsokefalou M, Chryssochoidis G. A theoretical and empirical investigation of nutritional label use. The European Journal of Health Economics. 2008;9:293–304. doi: 10.1007/s10198-007-0077-y 17924154

[39] Geiger CJ, Wyse BW, Parent CM, Hansen RG. Review of nutrition labeling formats. Journal of the American Dietetic Association. 1991;91(7):808–12. 2071796

[40] Kreuter MW, Brennan LK, Scharff DP, Lukwago SN. Do nutrition label readers eat healthier diets? Behavioral correlates of adults’ use of food labels. American journal of preventive medicine. 1997;13(4):277–83. 9236964

[41] Hager MH, Geiger C, Hill LJ, Martin C, Weiner S, Chianchiano D. Usefulness of nutrition facts label for persons with chronic kidney disease. Journal of renal Nutrition. 2009;19(3):204–10. doi: 10.1053/j.jrn.2009.01.014 19393919

[42] Variyam JN, Cawley J. Nutrition labels and obesity. National Bureau of Economic Research Cambridge, Mass., USA; 2006.

[43] Fitzgerald N, Damio G, Segura-Pérez S, Pérez-Escamilla R. Nutrition knowledge, food label use, and food intake patterns among Latinas with and without type 2 diabetes. Journal of the American Dietetic Association. 2008;108(6):960–7. doi: 10.1016/j.jada.2008.03.016 18502226

[44] An R. Diabetes diagnosis and nutrition facts label use among US adults, 2005–2010. Public health nutrition. 2016;19(12):2149–56. doi: 10.1017/S1368980015003079 26483168 PMC10270832

[45] Elfassy T, Yi S, Eisenhower D, Lederer A, Curtis CJ. Use of sodium information on the nutrition facts label in New York City adults with hypertension. Journal of the Academy of Nutrition and Dietetics. 2015;115(2):278–83. doi: 10.1016/j.jand.2014.08.027 25441962

[46] Gerber M. Biofactors in the Mediterranean diet. 2003. 10.1515/CCLM.2003.153 12964804

[47] Serra-Majem L, Ribas L, García A, Pérez-Rodrigo C, Aranceta J. Nutrient adequacy and Mediterranean Diet in Spanish school children and adolescents. European journal of clinical nutrition. 2003;57(1):S35–S9. doi: 10.1038/sj.ejcn.1601812 12947450

[48] Kanerva N, Kaartinen NE, Schwab U, Lahti-Koski M, Männistö S. The Baltic Sea Diet Score: a tool for assessing healthy eating in Nordic countries. Public health nutrition. 2014;17(8):1697–705. doi: 10.1017/S1368980013002395 24172174 PMC10282237

[49] Kim M, Yang J. Effects of nutrition claims on consumers’ health believes, brand attitudes and perceptions of disease-related risks. J consum stud. 2010;21:53–81.

[50] Jeong D-u Lee H-O, Kim Y-K Om A-S. A study on vitamin C content of nutrition emphasized products. Korean Journal of Community Nutrition. 2016;21(6):574–9.

[51] Jeong D-U, Lee H-O, Kim Y-K, Seo K-H, Om A-S. Minerals (calcium, iron, zinc) analysis and interaction of emphasized nutrition indication on products. Journal of Food Hygiene and Safety. 2016;31(6):420–5.

[52] Silayoi P, Speece M. Packaging and purchase decisions: An exploratory study on the impact of involvement level and time pressure. British food journal. 2004;106(8):607–28.

[53] Knowledge Misra R., attitudes, and label use among college students. Journal of the American Dietetic Association. 2007;107(12):2130–4. 18060900 10.1016/j.jada.2007.09.001

[54] Rothman RL, Housam R, Weiss H, Davis D, Gregory R, Gebretsadik T, et al. Patient understanding of food labels: the role of literacy and numeracy. American journal of preventive medicine. 2006;31(5):391–8. doi: 10.1016/j.amepre.2006.07.025 17046410

[55] Levy AS, Fein SB, Schucker RE. Performance characteristics of seven nutrition label formats. Journal of Public Policy & Marketing. 1996;15(1):1–15.

[56] Signal L, Lanumata T, Robinson J-A, Tavila A, Wilton J, Mhurchu CN. Perceptions of New Zealand nutrition labels by Māori, Pacific and low-income shoppers. Public Health Nutrition. 2008;11(7):706–13. 18167165 10.1017/S1368980007001395

[57] Kozup JC, Creyer EH, Burton S. Making healthful food choices: the influence of health claims and nutrition information on consumers’ evaluations of packaged food products and restaurant menu items. Journal of Marketing. 2003;67(2):19–34.

[58] Schucker RE, Levy AS, Tenney JE, Mathews O. Nutrition shelf-labeling and consumer purchase behavior. Journal of Nutrition Education. 1992;24(2):75–81.

[59] Drewnowski A. Concept of a nutritious food: toward a nutrient density score. The American journal of clinical nutrition. 2005;82(4):721–32. doi: 10.1093/ajcn/82.4.721 16210699

[60] Drewnowski A, Fulgoni V, Young M, Pitman S. Nutrient‐Rich Foods: Applying Nutrient Navigation Systems to Improve Public Health. Journal of Food Science. 2008;73(9):H222–H8. doi: 10.1111/j.1750-3841.2008.00963.x 19021805

[61] Rowlands JC, Hoadley JE. FDA perspectives on health claims for food labels. Toxicology. 2006;221(1):35–43. doi: 10.1016/j.tox.2005.10.023 16480811

[62] Fulgoni VL III, Keast DR, Drewnowski A. Development and validation of the nutrient-rich foods index: a tool to measure nutritional quality of foods. The Journal of nutrition. 2009;139(8):1549–54. doi: 10.3945/jn.108.101360 19549759

[63] Miller GD, Drewnowski A, Fulgoni V, Heaney RP, King J, Kennedy E. It is time for a positive approach to dietary guidance using nutrient density as a basic principle. The Journal of nutrition. 2009;139(6):1198–202. doi: 10.3945/jn.108.100842 19339707

[64] Wang X, Shi J, Kong H. Online health information seeking: a review and meta-analysis. Health Communication. 2021;36(10):1163–75. doi: 10.1080/10410236.2020.1748829 32290679

[65] Zakar R, Iqbal S, Zakar MZ, Fischer F. COVID-19 and health information seeking behavior: digital health literacy survey amongst university students in Pakistan. International Journal of Environmental Research and Public Health. 2021;18(8):4009. doi: 10.3390/ijerph18084009 33920404 PMC8069684

[66] Ebrahim A, Saif Z, Buheji M, AlBasri N, Al-Husaini F, Jahrami H. COVID-19 information-seeking behavior and anxiety symptoms among parents. OSP Journal of Health Care and Medicine. 2020;1(1):1–9.

[67] Ali A, Sohaib M, Iqbal S, Hayat K, Khan AU, Rasool MF. Evaluation of COVID-19 disease awareness and its relation to mental health, dietary habits, and physical activity: a cross-sectional study from Pakistan. The American journal of tropical medicine and hygiene. 2021;104(5):1687. doi: 10.4269/ajtmh.20-1451 33690156 PMC8103453

[68] Aslani N, Lazem M, Mahdavi S, Garavand A. A review of mobile health applications in epidemic and pandemic outbreaks: Lessons learned for COVID-19. Archives of Clinical Infectious Diseases. 2020;15(4).

[69] Grabia M, Markiewicz-Żukowska R, Puścion-Jakubik A, Bielecka J, Nowakowski P, Gromkowska-Kępka K, et al. The nutritional and health effects of the COVID-19 pandemic on patients with diabetes mellitus. Nutrients. 2020;12(10):3013. doi: 10.3390/nu12103013 33008059 PMC7600117

[70] Dimassi H, Haddad R, Awada R, Mattar L, Hassan HF. Food shopping and food hygiene related knowledge and practices during the COVID-19 pandemic: The case of a developing country. Italian Journal of Food Safety. 2021;10(2). doi: 10.4081/ijfs.2021.9384 34497780 PMC8404529

[71] O’Hara JK, Woods TA, Dutton N, Stavely N. COVID-19’s impact on farmers market sales in the Washington, DC, area. Journal of Agricultural and Applied Economics. 2021;53(1):94–109.

[72] Goldberg JH, Probart CK, Zak RE. Visual search of food nutrition labels. Human Factors. 1999;41(3):425–37. doi: 10.1518/001872099779611021 10665210

[73] Burton S, Garretson JA, Velliquette AM. Implications of accurate usage of nutrition facts panel information for food product evaluations and purchase intentions. Journal of the Academy of Marketing science. 1999;27:470–80.

[74] Howlett E, Burton S, Kozup J. How modification of the nutrition facts panel influences consumers at risk for heart disease: the case of trans fat. Journal of Public Policy & Marketing. 2008;27(1):83–97.

[75] Lin C-TJ, Lee J-Y, Yen ST. Do dietary intakes affect search for nutrient information on food labels? Social Science & Medicine. 2004;59(9):1955–67. 10.1016/j.socscimed.2004.02.030 15312929

[76] Institute KHID. National health and nutrition statistics 2020. Available from: https://www.khidi.or.kr/kps/dhraStat/result5?menuId=MENU01657&gubun=area&year=2020.

[77] Kalmpourtzidou A, Eilander A, Talsma EF. Global vegetable intake and supply compared to recommendations: a systematic review. Nutrients. 2020;12(6):1558. doi: 10.3390/nu12061558 32471188 PMC7352906

[78] King JC. Zinc: an essential but elusive nutrient. The American journal of clinical nutrition. 2011;94(2):679S–84S. doi: 10.3945/ajcn.110.005744 21715515 PMC3142737

[79] Annweiler C, Schott A-M, Rolland Y, Blain H, Herrmann F, Beauchet O. Dietary intake of vitamin D and cognition in older women: a large population-based study. Neurology. 2010;75(20):1810–6. doi: 10.1212/WNL.0b013e3181fd6352 21079183

[80] Vural Z, Avery A, Kalogiros DI, Coneyworth LJ, Welham SJ. Trace mineral intake and deficiencies in older adults living in the community and institutions: a systematic review. Nutrients. 2020;12(4):1072. doi: 10.3390/nu12041072 32294896 PMC7230219

[81] Borgmeier I, Westenhoefer J. Impact of different food label formats on healthiness evaluation and food choice of consumers: a randomized-controlled study. BMC public health. 2009;9(1):1–12. doi: 10.1186/1471-2458-9-184 19523212 PMC2702386

[82] Jones G, Richardson M. An objective examination of consumer perception of nutrition information based on healthiness ratings and eye movements. Public health nutrition. 2007;10(3):238–44. doi: 10.1017/S1368980007258513 17288620

[83] Van Kleef E, Van Trijp H, Paeps F, Fernandez-Celemin L. Consumer preferences for front-of-pack calories labelling. Public health nutrition. 2008;11(2):203–13. doi: 10.1017/S1368980007000304 17601362 PMC2811400

